# Structural Basis of Specific Recognition of Non-Reducing Terminal N-Acetylglucosamine by an *Agrocybe aegerita* Lectin

**DOI:** 10.1371/journal.pone.0129608

**Published:** 2015-06-26

**Authors:** Xiao-Ming Ren, De-Feng Li, Shuai Jiang, Xian-Qing Lan, Yonglin Hu, Hui Sun, Da-Cheng Wang

**Affiliations:** 1 National Laboratory of Biomacromolecules, Institute of Biophysics, Chinese Academy of Sciences, 15 Datun Road, Beijing, 100101, People’s Republic of China; 2 University of Chinese Academy of Sciences, No. 19A Yuquan Road, Beijing, 100049, People’s Republic of China; 3 College of Life Sciences, Wuhan University, Wuhan, 430072, People’s Republic of China; Indian Institute of Science, INDIA

## Abstract

O-linked N-acetylglucosaminylation (O-GlcNAcylation) is a reversible post-translational modification that plays essential roles in many cellular pathways. Research in this field, however, is hampered by the lack of suitable probes to identify, accumulate, and purify the O-GlcNAcylated proteins. We have previously reported the identification of a lectin from the mushroom *Agrocybe aegerita*, i.e., *Agrocybe aegerita* lectin 2, or AAL2, that could bind terminal N-acetylglucosamine with higher affinities and specificity than other currently used probes. In this paper, we report the crystal structures of AAL2 and its complexes with GlcNAc and GlcNAcβ1-3Galβ1-4GlcNAc and reveal the structural basis of GlcNAc recognition by AAL2 and residues essential for the binding of terminal N-acetylglucosamine. Study on AAL2 may enable us to design a protein probe that can be used to identify and purify O-GlcNAcylated proteins more efficiently.

## Introduction

Lectins are a large group of non-enzyme proteins that recognize and specifically bind to carbohydrate moieties that are either free or conjugated on the surfaces of proteins or lipids [1]. They are distinct from antibodies and mono- or oligosaccharide sensors/transporters. Lectins are ubiquitous in nature. They have been found in all kingdoms of organisms ranging from virus, bacteria, to eukaryotes including fungi, plants and animals [2]. Lectins play crucial roles in diverse biological processes at both molecular and cellular levels [1], such as cell-cell interactions, host-pathogen recognitions, and innate immune responses and metastasis [3]. Because of their capabilities to specifically recognize a highly diverse array of sugar moieties, lectins have been widely used in glycan studies: immobilized lectins, for example, have been invaluable tools in the enrichments and purifications of glycoproteins.

O-linked addition of N-acetylglucosamine (O-GlcNAcylation) on the serine and threonine residues of cytosolic and nuclear proteins is a reversible post-translational modification that plays essential cellular roles. Recent studies have shown that O-GlcNAcylation has extensive cross talk with phosphorylation, another essential protein modification, and serves as a nutrient sensor for the modulation of numerous signaling and transcription pathways [4, 5]. It was observed that major oncogenic factors such as p53, c-Myc, NFκB, and β-catenin are O-GlcNAcylated and that chromatin is modulated by O-GlcNAcylation, indicating that this modification may also serve as a cancer hallmark [6]. O-GlcNAcylation is distinct from other types of glycosylations in that it only adds one GlcNAc, a small, non-charged moiety, onto the serine/threonine residues, resulting in a modification that is very difficult to detect using techniques such as 2-dimensional electrophoresis; the modification is also too labile to be readily detected by conventional mass spectroscopies [4, 7]. The lack of efficient probes led to the discovery of O-GlcNAcylation only in early 1980s [8], decades later than that of other protein glycosylations, despite its ubiquitous presence in eukaryotic cells. This lack of efficient tools still hampers the research in this important field [4, 7].

In this paper, we report the crystal structures of a lectin from *Agrocybe aegerita*, designated as AAL2, in its apo- and complex forms with GlcNAc and GlcNAcβ1-3Galβ1-4GlcNAc, as part of our endeavors to develop a system to facilitate the identification and purification of O-GlcNAcylated proteins.

Lectins from fungi (mainly from mushrooms) have attracted wide attention in recent years owing to their diverse bioactivities [9], including antitumor, antiproliferative, and immunomodulatory activities [10–12]. AAL2 was recently discovered from the fruit body of the edible mushroom *A*. *aegerita*. It consists of 407 amino acid residues, with a molecular weight of 43.17 kDa. It was the second lectin purified from *A*. *aegerita* to be characterized by x-ray crystallography by our groups, after *A*. *aegerita* AAL, an antitumor lectin that recognizes and binds Thomsen-Friedenreich antigen [13]. AAL2 was found to specifically bind nonreducing terminal N-acetylglucosamine (GlcNAc) moieties of either alpha- or beta-linkage and was reported to have the activity of inducing apoptosis in hepatoma cells *in vitro* as well as inhibiting hepatoma growth in tumour-bearing mice *in vivo* [14]. Glycan array analysis demonstrated that out of 465 glycans tested, AAL2 has a unique preference for GlcNAc-(Gal-GlcNAc)_1–3_ over (Gal-GlcNAc)_1–3_, which lacks the terminal GlcNAc [14]. ITC assays also revealed that AAL2 has higher affinity towards GlcNAc compared to WGA (wheat germ agglutinin) and GSL-II (*Griffonia simplicifolia* lectin-II), which have been widely used in glycan studies for their GlcNAc-binding activities [15, 16]. AAL2, therefore, has the potential to be a better tool for research involving O-GlcNAcylated proteins.

To illustrate the mechanisms of how AAL2 specifically recognizes non-reducing terminal GlcNAc, we performed structural and surface plasmon resonance (SPR) affinity characterizations on AAL2 and its binding with GlcNAc and GlcNAcβ1-3Galβ1-4GlcNAc, as well as its divalent metal cation binding properties. These results showed that AAL2 has homologous sequence and similar structure with previously characterized fungal lectins PVL (*Psathyrella velutina* Lectin) [17], and it exclusively recognizes glycans with non-reducing terminal GlcNAc and the binding affinity between AAL2 and GlcNAc is relatively high. Our work, therefore, lays a solid foundation for the development of an efficient macromolecule probe that can be used to identify, enrich, and purify O-GlcNAcylated proteins and to locate the sites of modification.

The crystal structure of PVL, a fungal lectin from *Psathyrella velutina*, was reported in 2006 [17], which was the first member of the seven-blade β-propeller family of fungal lectins to be structurally characterized. But the specific recognition mechanism of glycans by the lectin protein needed further understanding. AAL2 is homologous with PVL and belongs to the same lectin family. To illustrate the mechanisms of how AAL2 specifically recognizes non-reducing terminal GlcNAc, we performed structural and SPR affinity characterizations on AAL2 and its binding with GlcNAc and GlcNAcβ1-3Galβ1-4GlcNAc, as well as its divalent metal cation binding properties. The results revealed the main structural basis for the exclusive recognition between AAL2 and glycans with non-reducing terminal GlcNAcs. Structure comparison showed a rather high similarity between AAL2 and PVL, indicating that these two lectins adopt a unique structure to specifically recognize glycans with non-reducing terminal GlcNAc. Our present work, in association with previous information, therefore, lays a solid foundation for the development of an efficient macromolecule probe that can be used to identify, enrich, and purify O-GlcNAcylated proteins and to locate the sites of modification.

## Materials and Methods

### Purification and crystallization

The gene of *AAL2* was amplified from *A*. *aegerita* and cloned into pET30a expression vector between *NdeI* and *HindIII* restriction sites, without any tag. The recombined plasmid was transformed into *Escherichia coli* host strain BL21 (DE3) competent cells for expression. The AAL2 protein expression and first-step purification with a GlcNAc-coupled Sepharose 6B affinity column has been described in detail previously [14, 18]. The protein samples were then applied to a HiTrap SP XL column (GE Healthcare Life Science) to remove the glycan ligands that may bind AAL2. A Superdex 200 10/300 GL gel-filtration column (Amersham Bio-sciences) was subsequently used to further purify and to identify the oligomeric state. The purified protein was concentrated to 30 mg/ml in a buffer containing 50mM Tris-HCl pH 7.5 and 150mM NaCl.

AAL2 was mixed with GlcNAc (from Sigma) and GlcNAcβ1-3Galβ1-4GlcNAc trisaccharide (a gift from Consortium for Functional Glycomics), respectively, at AAL2:ligand molar ratios of 1:10, and the mixtures as well as pure AAL2 sample were used in crystallization condition searches. Crystallization was performed using the hanging-drop vapor diffusion method, by mixing protein and reservoir solutions with equal volume of 1μl and equilibrated against 400μl reservoir solutions at 293K. Crystals of AAL2 were obtained under the condition of 30% PEG 8000 (w/v), 0.2M sodium acetate and 0.1M sodium cacodylate pH 6.4. Crystals of AAL2-GlcNAc complex were obtained with the solution containing 25% PEG 4000 (w/v), 6% Tacsimate pH 6.0 and 0.1M MES pH 6.0. The crystals of AAL2-trisaccharide complex were obtained in solution containing 2.3M ammonium sulfate and 0.1M sodium acetate pH 4.4.

### Data collection, structural solution and refinement

All the three types of crystals were mounted on cryoloops (Hampton Research) directly from the crystallization drops and flash-cooled in a nitrogen-gas stream at 95 K. X-ray diffraction data for AAL2 was collected on an in-house FR-E Bluemax/R-AXIS IV++/Varimax HF (Rigaku) diffractometer at wavelength of λ = 1.5418Å; the data for AAL2 complexed with GlcNAc was collected on beamline 1W2B at Beijing Synchrotron Radiation Facility (BSRF; Beijing, China) at λ = 1 Å; and the data for AAL2 complexed with GlcNAcβ1-3Galβ1-4GlcNAc was collected on an in-house MicroMax 007 Saturn944+ VarimaxHF (Rigaku) diffractometer at λ = 1.5418 Å. Each dataset was collected with a total of 180 images using an oscillation of 1° per image.

The data were indexed and integrated using the program *MOSFLM* v.7.0.4 [19] and then scaled by *SCALA* v.6.0 from the *CCP*4 v.6.0.2 program suite [20]. A sequence-homology BLAST search at NCBI website (http://www.ncbi.nlm.nih.gov/BLAST) identified a structurally characterized protein PVL that has 60% sequence identity with AAL2 [17]. The structure of AAL2 was solved using molecular replacement method as implemented in the *AutoMR* program of the Phenix suite v.1.8.2 [21] with the structure of PVL (PDB 2BWR) as a search model. The determination of the structures of the two complexes was similarly performed using *AutoMR* program in Phenix, with the structure of solved AAL2 as search models. The carbohydrate ligands were added and adjusted manually according to the electronic density map displayed in Coot [22]. The structural refinement was performed using the *Refine* program in Phenix alternated with manual rebuilding using the graphical program Coot.

### Surface plasmon resonance

The SPR experiments were carried out on a BIAcore T100 (GE Healthcare, Sweden) using CM5 sensor chips at 298 K. AAL2 protein solution was diluted to a concentration of 20μg/ml with fixing buffer (pH 5.0) and subsequently immobilized by amine coupling onto the chips. The immobilized level of AAL2 reached 3000 response units. Flow path 1 was used as an activated blank control for subtraction. Interaction experiments were performed in a running buffer of 50mM Tris-HCl pH 7.5, 150mM NaCl, 0.005% Tween 20 at a flow rate of 30μl/min. The ligands in sample buffer were employed as the flowing phase respectively. Each interaction experiment was carried out with gradient concentrations from low to high programmed to inject at a 30μl/min flow rate under a sensorgram. Binding curves were corrected, aligned and fitted kinetically using the BIAcore T100 evaluation program (GE Healthcare).

### Determination of metal binding properties

The purified AAL2 was loaded on a HiTrap Desalting Column (GE Healthcare) with buffer containing 50mM Tris-HCl pH 7.5, 150mM NaCl, and 5mM EDTA to remove metal ions from the protein. The flow-through fraction of protein was incubated in 277K for 1 hr. and then loaded on a HiTrap Desalting Column once again with the buffer of 50mM Tris-HCl pH 7.5 and 150mM NaCl to remove EDTA and EDTA-coupled metal ions. The flow-through fraction of protein was then evenly divided into five parts, and was added solutions of MgCl_2_, CaCl_2_, MnCl_2_, CoCl_2_, and NiSO_4_, respectively, to concentrations of 10mM. All the samples were incubated at 277K overnight and then applied to a HiTrap Desalting Column again to remove free metal ions. The metal content of the five samples as well as the protein solution not processed with EDTA were determined on a Varian Inductively Coupled Plasma—Atomic Emission Spectrometry (ICP-AES) (Agilent Technologies, USA). SPR experiments were also performed on EDTA-treated sample in buffers containing 5mM CaCl_2_ to find out if the metal binding state of AAL2 would affect its affinity to ligands.

## Results

### GlcNAc is the most effective ligand for AAL2

**Fig 1:**
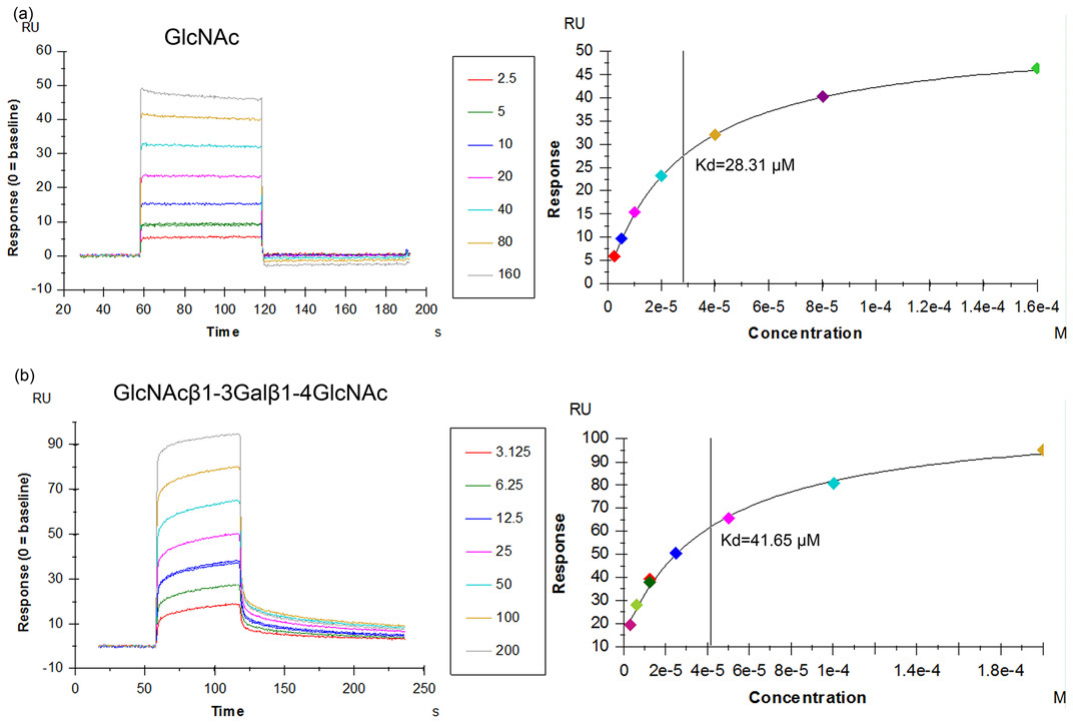
SPR analysis of binding of GlcNAc and GlcNAcβ1-3Galβ1-4GlcNAc to immobilized AAL2 at 298 K. (a) The binding curves measured at different concentrations of GlcNAc and the steady state affinity indicated by the value of Kd. (b) The binding curves measured at different concentrations of GlcNAcβ1-3Galβ1-4GlcNAc and the steady state analysis shows that the affinity of AAL2 to the trisaccharide is close to but a little lower than that to GlcNAc.

To measure the affinities between AAL2 and glycan ligands, we performed Surface Plasmon Resonance (SPR) experiments. The ligands we used were GlcNAc monosaccharide and GlcNAcβ1-3Galβ1-4GlcNAc, a trisaccharide showing very high affinity toward AAL2 in glycan array analysis. In SPR experiments, the experimental curves obtained automatically from different sensorgrams showed that both GlcNAc and GlcNAcβ1-3Galβ1-4GlcNAc could be captured efficiently by the sensor chip surface through binding to AAL2 and quickly dissociated to the baseline level (Fig 1). The data obtained from the analysis of stable-state affinity model showed that AAL2 binds to GlcNAc with a dissociation constant (Kd) of 28.31 μM (Fig 1a), while previous assays using ITC techniques obtained a Kd value of 195μM [14], which was comparable to other GlcNAc-binding fungal lectins such as PVL (*Psathyrella velutina* Lectin) (Kd = 214 μM) [17] and BLL (Boletopsis leucomelas lectin) (Kd = 435 μM) [23]. AAL2 was also shown via SPR to bind to the trisaccharide with a Kd value of 41.65 μM, which is close to that of GlcNAc (Fig 1b), demonstrating that Gal-GlcNAc moiety beyond GlcNAc may not affect the binding between AAL2 and trisaccharide.

### Overall structures of AAL2 and AAL2-glycan complexes

The structures of AAL2, AAL2-GlcNAc and AAL2-GlcNAcβ1-3Galβ1-4GlcNAc were determined at resolutions of 2.0Å, 2.1Å, and 2.0Å, respectively. The data collection and refinement statistics are summarized in Table 1.

**Table 1:** Data collection, processing and structural refinement statistics for AAL2, AAL2 complex with GlcNAc and AAL2 complex with GlcNAcβ1-3Galβ1-4GlcNAc.

	AAL2	AAL2 with GlcNAc	AAL2 with GlcNAcβ1-3Galβ1-4GlcNAc
*Data collection*			
Space group	P 2_1_ 2_1_ 2_1_	P 2_1_ 2_1_ 2_1_	P 2 2_1_ 2_1_
Cell dimensions			
a(Å)	52.60	52.56	54.23
b(Å)	111.70	111.24	77.54
c(Å)	135.97	134.63	89.02
Wavelength(Å)	1.54187	1.00001	1.54187
Subunits/asym.	2	2	1
Resolution range(Å)	51.66–2.00	26.93–2.10	46.31–2.00
High resolution shell(Å)	2.11–2.00	2.21–2.10	2.11–2.00
Matthews coefficient	2.29	2.18	2.18
Solvent content(%)	45.84	46.28	55.72
*R*_merge_ (%)^a^	5.7(27.6)	14.1(41.0)	12.7(38.3)
Average *I*/σ*I* ^a^	16.8(3.8)	6.3(2.2)	10.2(2.9)
Completeness^a^	95.7(87.9)	88.8(70.4)	98.8(92.1)
Redundancy^a^	5.0(3.0)	3.0(1.8)	6.2(3.4)
*Refinement*			
No. reflections	612304	350907	334833
*R*_work_/*R*_free_ (%)	18.71/23.49	17.17/24.08	19.02/24.17
Average B-factors(Å^2^)	21.95	11.38	23.89
*R*.*m*.*s*. *deviations*			
Bond lengths(Å)	1.050	1.093	0.758
Bond angles(°)	0.007	0.007	0.003
Ramachandran outliers	0.1%	0.1%	0
Most favored region (%)	97.1	95.6	96.5

*R*_*merge*_ = Σ_*hkl*_Σ_*i*_|*I*_*i*_(*hkl*)-⟨*I*(*hkl*)⟩|/Σ_*hkl*_Σ_*i*_ *I*_*i*_(*hkl*), where *I*_*i*_(*hkl*) is the intensity of the measurement of reflection *hkl* and ⟨*I*(*hkl*)⟩ is the mean value of *I*_*i*_(*hkl*) for all *i* measurements.

^a^The values in parentheses refer to the highest resolution shell.

There are two virtually identical AAL2 monomers arranged around a two-fold pseudoaxis of symmetry in an asymmetric unit in both the structures of AAL2 and AAL2-GlcNAc, and one AAL2 monomer in AAL2-GlcNAcβ1-3Galβ1-4GlcNAc, while the protein exists as monomers in solution as determined from its elution volume on a Superdex 200 10/300 GL column. Discussions thereafter will be focused on monomer A unless specified otherwise.

As expected from the sequence homology, the structures of AAL2 and AAL2-GlcNAc complex are very similar to those of the corresponding structures of PVL, with root-mean-square deviations (RMSDs) of 0.7Å for Cα atoms.

#### AAL2 adopts a beta-propeller fold

**Fig 2:**
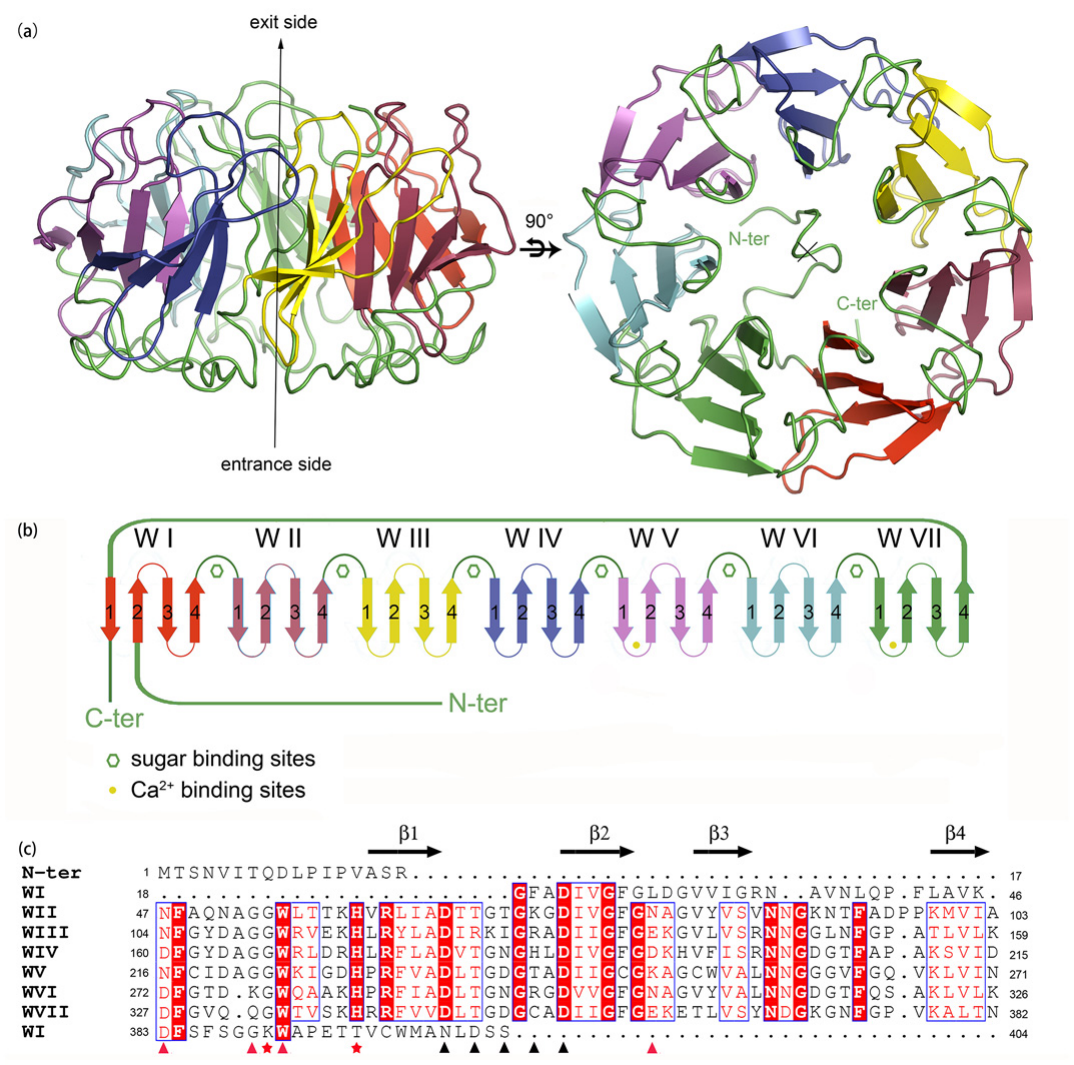
The overview of the structure of AAL2. (a) Two orthogonal views of the crystal structure of native AAL2. (b) Topology diagram of AAL2 shows that the seven blades have a shifting of one β-strand compared with the seven sequence repeats. GlcNAc binding sites and calcium binding sites are represented by green hexagons and solid yellow circles. (c) Alignment of the seven W-motifs of AAL2. Solid red triangles, black triangles, and red pentagons indicate the residues forming polar interactions with GlcNAc, the residues coordinate calcium ions, and the differences between WI and other W-motifs making it incapable of binding GlcNAc, respectively.

The overall structure of AAL2 monomer is a single domain with the shape of cylindrical torus with a diameter of about 51Å and a height of about 27Å, showing a very regular, seven-bladed β-propeller fold formed by the blades packing in a circular array (Fig 2a). Each blade is a β-sheet consisting of four anti-parallel β-strands arranged in a W-like shape and of about 56 residues in length, and is named W-motif according to its shape. Seven tandem W-motifs of the AAL2 monomer, numbered WI to WVII from N- to C-terminus (Fig 2b), are organized around a seven-fold axis of pseudo symmetry.

The four β-strands of each W-motif are numbered 1–4 according to their spatial arrangement from AAL2 center to its surface (Fig 2b). The first β-strands of the seven W-motifs are located innermost of the AAL2 monomer and form a central tunnel that is filled with water molecules and almost blocked by a long N-terminus loop (except for a small opening). These 7 innermost strands point to the same direction, from the ‘entrance’ to the ‘exit’ of the central tunnel, and run almost parallel to each other along the direction of the seven-fold propeller axis (Fig 2a). Strands 1 and 2 are connected by a long loop of about eight residues, while strands 2 and 3 are connected by a short loop of only four residues (except for a β-turn in the first W-motif). Strands 3 and 4 are connected by a long loop of about eleven residues. After strand 4, the peptide chain goes towards the direction of the central tunnel through the large ‘connecting segment’ to start the strand 1 of next β-sheet. When the AAL2 molecule is viewed along the central 7-fold symmetry axis, all the seven ‘blades’ are twisted (Fig 2a right panel), with each β-strand twists about 30° clockwise compared to the prior one, resulting in the arrangements of the outmost β-strands as almost perpendicular to the propeller axis and form a outer circular edge of the propeller between themselves. This arrangement may help stabilize the protein structure. Sequence alignment (Fig 2c) and superposition of the seven W-motifs show that they have high homologies to each other.

What is notable is that the seven sequence repeats observed in AAL2 do not match exactly with the seven blades, but instead have a shifting of one β-strand (Fig 2b). WI consists of three consecutive strands, i.e., strands 2, 3 and 4 at the N-terminus, and one strand, i.e., strand 1, from the C-terminus. This property of ‘end to end’, which has been discovered in many propeller structures, has a great evolutionary advantage of stabilizing protein structures.

### The carbohydrate binding sites

The structure of AAL2-GlcNAc complex revealed six binding sites out of the seven W-motifs, with the exception of motif WI. All the bound GlcNAc molecules have virtually identical orientations in the structure.

**Fig 3:**
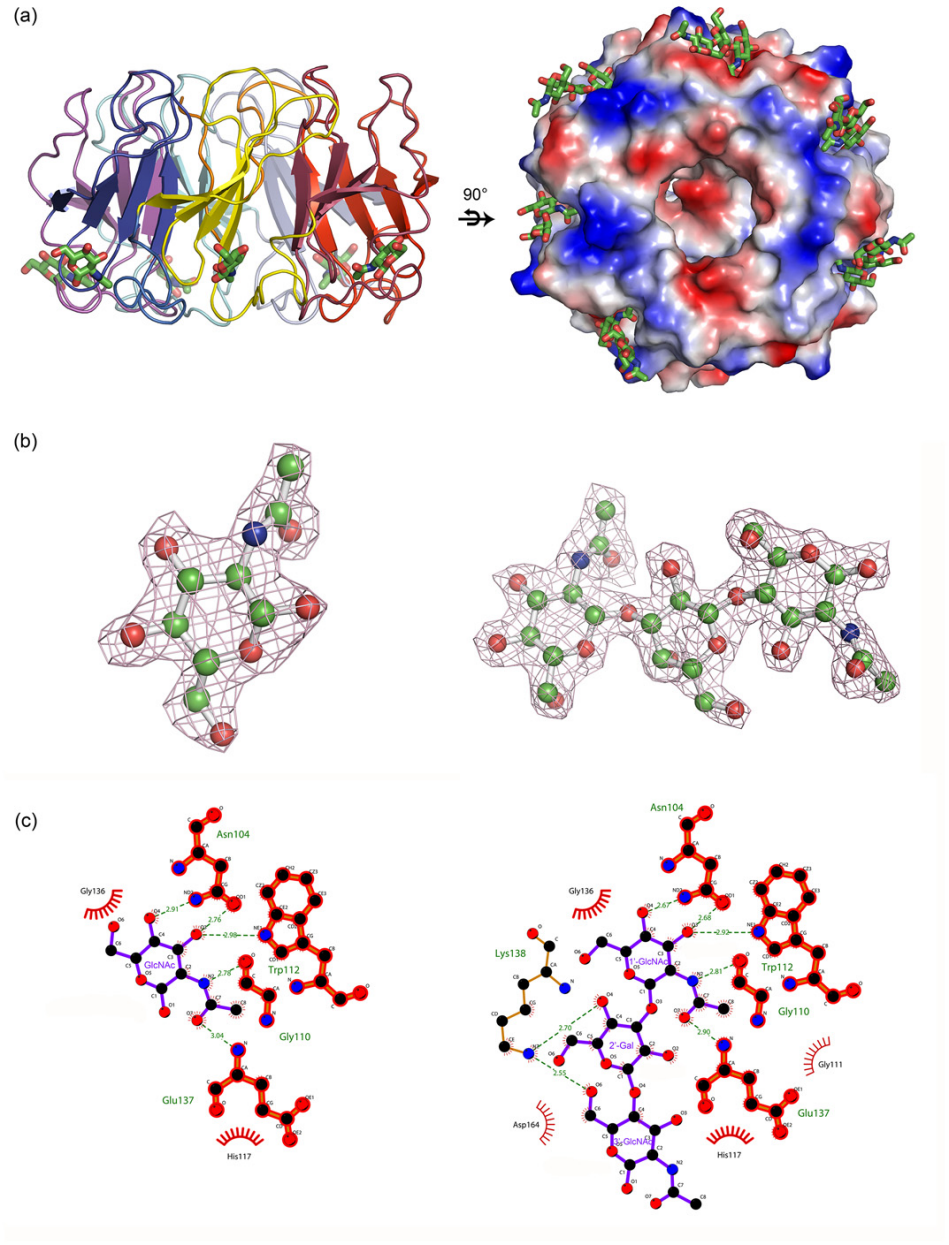
Overview of the six glycan binding pockets of AAL2, alignment of the six GlcNAc molecules and the detailed interaction in WIII. (a) Horizontal view of AAL2-GlcNAc complex (left) and the surface electrostatic potential diagram of the vertical view of AAL2- GlcNAcβ1-3Galβ1-4GlcNAc complex (right). (b) Sigma-A weighted *Fo-Fc* omit electron density map around GlcNAc (left) and GlcNAcβ1-3Galβ1-4GlcNAc (right). (c) The hydrogen bonds and hydrophobic interactions between AAL2 and GlcNAc (left) and GlcNAcβ1-3Galβ1-4GlcNAc (right), respectively, shown in Ligplot.

Glycan ligands are bound in shallow pockets located on the entrance side, between the connecting segment and the 2–3 loop of each W-motifs (except for WI) (Fig 3a). Electron density maps show that the two ligands in the complex are well ordered and all the carbohydrate rings in the ligands adopt chair conformation (Fig 3b). Both hydrogen bonds and hydrophobic interactions contribute to the binding of AAL2 with the ligands (Fig 3c).

#### AAL2-GlcNAc recognition pockets

Because the six binding pockets of AAL2 for glycan ligands are almost identical, we chose motif WIII for detailed discussion and the descriptions also applied to other W-motifs unless specified otherwise. The carbohydrate binding pockets of AAL2 are very similar to those of PVL, as implied by the sequence homology.

**Fig 4:**
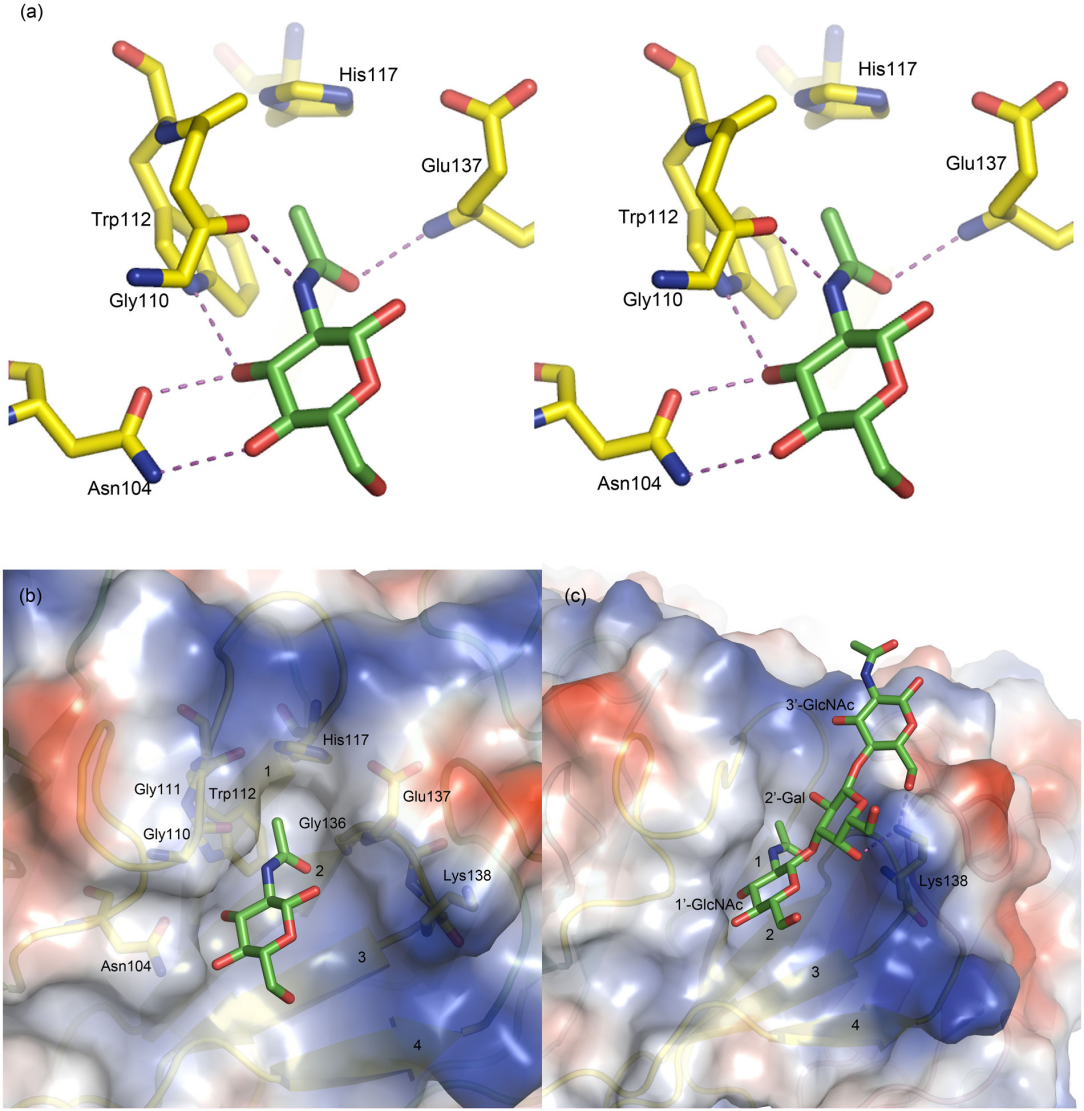
(a) Stereo view of GlcNAc bound to the binding pocket in WIII. (b) (c) Surface electrostatic potential diagrams of the binding pockets in WIII for GlcNAc and GlcNAcβ1-3Galβ1-4GlcNAc, respectively.

The binding pocket for GlcNAc consists of two parts. The major part is formed by the segment connecting WII and WIII, including the side chains of Asn104, Trp112 and His117 and main chain atoms of Gly110 and Gly111. The other part is formed by the main chain atoms of Gly136, Glu137, and Lys138 from the 2–3 loop of WIII. Among the residues mentioned above, Asn104 is located at the beginning of the connecting segment and it is either Asn or Asp in the corresponding position of each blade, and offers its OD1 and ND2/OD2 to form hydrogen bonds with O3 and O4 hydroxyl groups of GlcNAc, respectively. The O3 hydroxyl of GlcNAc moiety also forms a hydrogen bond with NE1 of Trp112. The N-acetyl group of GlcNAc forms two hydrogen bonds with the main chain of the protein: N2 donates one to O of Gly110, and O7 carbonyl accepts one from N of Glu137 (Fig 4a). The hydrogen bonding networks between AAL2 and GlcNAc of all six binding sites are listed in Table 2. Furthermore, the inner part of the pocket comprised of Trp112, His117, Gly136 and Glu137 is hydrophobic, helping stabilize the complex structures (Fig 4b). With the presence of both the polar and hydrophobic interactions, GlcNAc is bound tightly in the shallow pocket formed on the protein surface, with the head of N-acetyl group buried inside the pocket and the reduced O1 as well as O6 hydroxyl pointing outside into the solvent region.

**Table 2:** Polar interactions (distances in Å) between atoms from GlcNAc (left column) and AAL2.

	WII		WIII		WIV		WV		WVI		WVII	
N2	G53[O]	2.79	G110[O]	2.78	G166[O]	3.17	G222[O]	2.78	K277[O]	2.74	Q332[O]	3.12
O7	N80[N]	2.89	E137[N]	3.04	D193[N]	2.94	K249[N]	2.98	N304[N]	2.87	E359[N]	2.77
O3	N47[OD1]	2.64	N104[OD1]	2.76	D160[OD1]	2.55	N216[OD1]	2.70	D272[OD1]	2.60	D327[OD1]	2.50
O3	W55[NE1]	2.85	W112[NE1]	2.98	W168[NE1]	2.80	W224[NE1]	2.95	W279[NE1]	2.88	W334[NE1]	2.79
O4	N47[ND2]	2.79	N104[ND2]	291	D160[OD2]	2.72	N216[ND2]	2.81	D272[OD2]	2.55	D327[OD2]	2.78
O6									Y308[OH]	2.56		

GlcNAcβ1-3Galβ1-4GlcNAc ligands are bound to the same pockets of AAL2, with the non-reducing terminal GlcNAc (1’-GlcNAc) adopted the same position as GlcNAc. The polar interactions between AAL2 and the trisaccharide are summarized in Table 3. The interactions between AAL2 and 1’-GlcNAc of the trisaccharide are identical to those with GlcNAc monosaccharide. Although there are several hydrogen bond interactions between AAL2, such as Lys138, and the LacNAc moiety after 1’-GlcNAc, AAL2 doesn’t demonstrate specific preference to the 2’-Gal or 3’-GlcNAc in all the six blades (Fig 4c), since the interactions seem to be random. This result was rather unexpected based on the glycan array. However, our results have further proved that AAL2 recognizes the terminal non-reducing GlcNAc moiety of a polysaccharide.

**Table 3:** Polar interactions (distances in Å) between GlcNAcβ1-3Galβ1-4GlcNAc and AAL2.

Binding site	WII		WIII		WIV		WV		WVI		WVII	
1’-GlcNAc												
N2	G53[O]	2.81	G110[O]	2.81	G166[O]	2.94	G222[O]	2.82	K277[O]	2.94	Q332[O]	2.89
O7	N80[N]	2.97	E137[N]	2.90	D193[N]	3.09	K249[N]	2.91	N304[N]	2.86	E359[N]	3.04
O3	N47[OD1]	2.72	N104[OD1]	2.68	D160[OD1]	2.61	N216[OD1]	2.72	D272[OD1]	2.62	D327[OD1]	2.69
O3	W55[NE1]	2.86	W112[NE1]	2.92	W168[NE1]	2.99	W224[NE1]	2.79	W279[NE1]	2.72	W334[NE1]	2.85
O4	N47[ND2]	2.85	N104[ND2]	2.67	D160[OD2]	2.64	N216[ND2]	2.90	D272[OD2]	2.78	D327[OD2]	2.95
2’-Gal												
O2	N80[ND2]	2.54			D193[OD1]	2.47	K249[NZ]	3.40				
O4			K138[NZ]	2.70							K360[OD2]	3.07
O5			K138[NZ]	2.66								
3’-GlcNAc												
O6			K138[NZ]	2.55								

#### Essential residues and structural elements for substrate recognition

**Fig 5:**
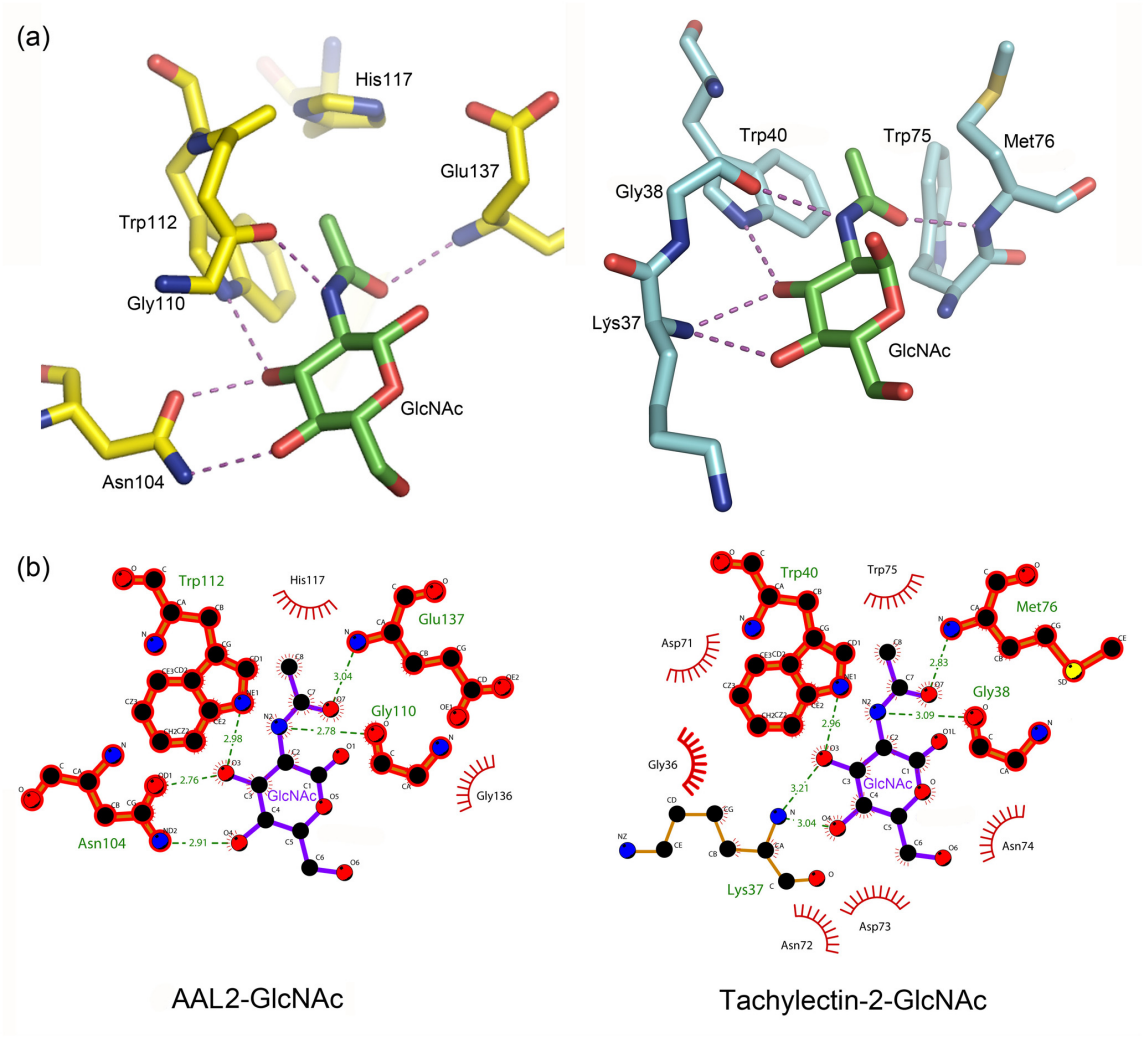
(a) The comparison of hydrogen bonds network between AAL2 (left) and Tachylectin-2 (right) and GlcNAc. (b) Both polar and hydrophobia interactions between AAL2 (left) and Tachylectin-2 (right) and GlcNAc. GlcNAc is shown in purple and the residues that are marked bold show the same components of the glycan binding pockets in the two lectins.

Sequence alignment of all seven W-motifs (Fig 2c) shows that the residues involved in the polar interactions with GlcNAc are highly conserved in all the seven blades. We have mutated five Asn104-counterpart residues in other W-motifs to alanine and found that the mutant proteins could not bind the GlcNAc-coupled Sepharose 6B affinity column. This showed that Asn104 and its counterparts in other W-motifs are essential for hydrogen bond interactions with GlcNAc. The Tachylectin-2 [24] from *Tachypleus tridentatus* binds GlcNAc in a manner almost identical to that of AAL2, although the binding sites of these two proteins do not share the same architecture (Fig 5). While residues Lys37 and Trp40 are responsible for the GlcNAc specificity of Tachylectin-2, their equivalent residues are Asn104 and Trp112, respectively. Several other residues contribute to hydrogen bond formation with the substrates with their main-chain atoms. Another important common element involved in GlcNAc-binding is a hydrophobic pocket for the accommendation of the acetyl group of GlcNAc, which is formed by two aromatic residues, *i*.*e*., residues Trp40 and Trp75 in Tachylectin and Trp112 and His117 in AAL2. We therefore propose that AAL2 represents a common mode of GlcNAc recognition, although more GlcNAc-binding lectin structures are needed to firmly establish the structural patterns.

#### Unique stereochemistry for AAL2 specific recognition

**Fig 6:**
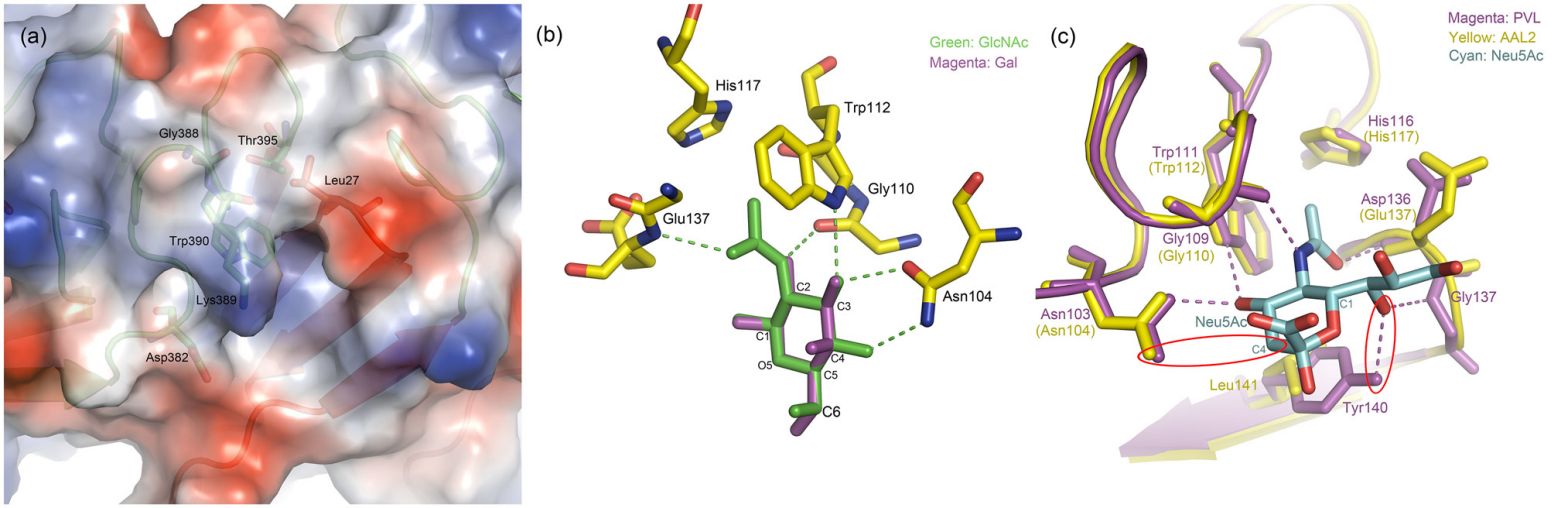
(a) Surface electrostatic potential diagram of WI that shows Lys395 blocked the potential glycan binding pocket. (b) Comparison between GlcNAc and Gal in conformation in the binding pocket of WIII from AAL2. (c) Superposition of the binding pockets in WIII from AAL2 (yellow) and PVL (magenta) shows that Neu5Ac (cyan) is not a specific ligand for AAL2.

The putative binding site of WI seems intact and should be able to bind one more GlcNAc. However, two differences rule out this possibility. Firstly, instead of glycine, it is a lysine at residue 389, with a relatively large side chain stretching towards the binding pocket and it would block the binding of GlcNAc to the protein (Fig 6a). Another difference is observed at residue 395, where a threonine residue replaces the conserved histidine residue, which, as mentioned above, contributes to the hydrophobic interaction in all other six W-motifs.

Mutation assays also explained why AAL2 has the specificity towards GlcNAc instead of GalNAc. Since galactose is a C-4 epimer of glucose, the O4 hydroxyl group in GlcNAc is equatorial (in the "plane" of the ring) while in GalNAc it is axial (up from the "plane" of the ring). If a Gal moiety were to take the position and orientation of GlcNAc as in the complex structures, the key hydrogen bonds between Asn104 and O4 hydroxyl of GlcNAc would be disrupted (Fig 6b). AAL2 did not show any binding affinities toward Neu5Ac as assayed by SPR, even though it shares 60% sequence identity and has very similar structure to those of PVL, which, in contrast, binds both GlcNAc and Neu5Ac. This lack of affinity towards Neu5Ac might be resulted from the fact that Neu5Ac does not have the O4 hydroxyl to form the key hydrogen bond with AAL2 as mentioned above. Moreover, the side chain of residue Tyr 140 of PVL provides the Neu5Ac specificity by forming a hydrogen bond with sialic acid moiety of Neu5Ac (Fig 6c), but its counterpart in AAL2, *i*.*e*., residue Leu141, is too small to form a similar hydrogen bond. The structural evidences prove that AAL2 has an exclusive specificity to non-reducing terminal GlcNAc which perfectly fits with the glycan array and the hydrogen bond donated by O4 hydroxyl group of GlcNAc is crucial for its binding to AAL2. In other words, the corresponding residues of Asn104 play a most important role in determining the glycan specificity of AAL2.

### Metal binding property of AAL2

**Fig 7:**
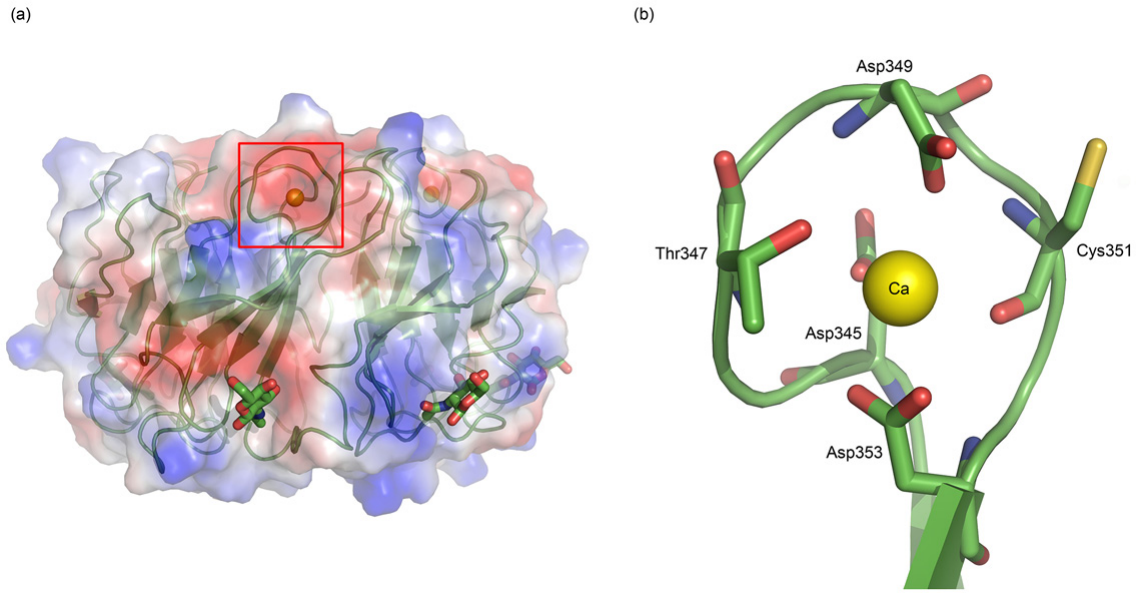
(a) Overview of the locations of two Ca^2+^, which are represented by yellow spheres, in the whole structure. (b) Larger and detailed view of the image section in red frame of (a).

In addition to glycans, AAL2 also binds two cations per molecule as observed in the structure of AAL2-GlcNAc complex (Fig 7a). Similar metal ion binding properties were also observed for PVL [17]. These cations are located in the 1–2 loops of WV and WVII of AAL2, where highly negatively-charged cages are formed (Fig 7b).

To identify the cation-binding preferences of AAL2, we tested Mg^2+^, Ca^2+^, Mn^2+^, Co^2+^and Ni^2+^for their binding affinities. The binding assay results are shown in Table 4. Because of heavy precipitation upon adding zinc salt into protein solution, the binding assay of Zn^2+^ was not shown. But there was indeed zinc in the purified native protein samples. Calcium is the main element AAL2 binds, which is the reason why we modeled cations in the AAL2-GlcNAc complex as Ca^2+^. The preference of the divalent cation type may lie in the diameter fitting the size of the negative charged pocket. SPR assay were also carried out and illustrated that the adding of Ca^2+^ did not affect the glycan affinity of AAL2. Although it has been reported that Ca^2+^ binding property is a common phenomenon observed in propeller structures and it was believed that the binding of Ca^2+^ play a role of stabilizing the overall structure [17], similar conclusions could not be made for AAL2 based on our studies. So the effect of metal binding of AAL2 remains to be investigated.

**Table 4:** Metal content of AAL2 samples prepared with various divalent metal ions.

	Mg^2+^	Ca^2+^	Mn^2+^	Zn^2+^	Cu^2+^	Co^2+^	Ni^2+^(mg/L)
AAL2+MgCl_2_	0.020						
AAL2+CaCl_2_		0.17					
AAL2+MnCl_2_			0.042				
AAL2+CoCl_2_						0.022	
AAL2+NiSO_4_							0.0068

## Discussions

In this study, we have demonstrated that AAL2 has relatively high affinities and exclusive specificity towards terminal nonreducing GlcNAc and illustrated the structural basis of its ligand specificity.

Although further studies are required to reveal the antitumor mechanisms of AAL2, the high affinities and exclusive specificity of this lectin toward nonreducing GlcNAc moieties strongly point to the possibilities that AAL2 may exert its functions through interactions with O-GlcNAcylated proteins. Many tumor suppressors and oncogenes that involved in transcription regulations, such as c-Myc, p53, and retinoblastoma, are modified by O-GlcNAcylation. The extensive cross talk between O-GlcNAcylation and phosphorylation involving these proteins underlines the roles played by O-GlcNAcylation in tumor pathogenicity [4, 6], but the exact mechanisms and ligand proteins of AAL2 antitumor functions remain to be revealed.

The studies reported here, on the other hand, have demonstrated that AAL2 is a very promising candidate to replace WGA in glycan research for the enrichment and purification of O-GlcNAcylated proteins. WGA lectin weak affinity chromatography has been the most frequently used method in the enrichment and purification of O-GlcNAcylated proteins [16], but this method suffers from, as implied by its name, the weak affinity of WGA toward its ligands and low specificities, because WGA binds sialic acids in addition to O-GlcNAcylated proteins. AAL2, in contrast, has higher affinities and exclusive specificities towards terminal GlcNAc moieties. Further research is in progress in our labs to employ AAL2 in affinity chromatography for the efficient enrichment and purification of O-GlcNAcylated peptides and proteins.

The results reported here illustrated the structural basis of how AAL2 recognizes and binds terminal GlcNAc moiety and laid a solid foundation for us to reveal the antitumor mechanisms of this lectin and to develop a better reagent for glycan studies involving O-GlcNAcylated proteins.
